# Interpersonal Violence Is Associated with Self-Reported Stress, Anxiety and Depression among Men in East-Central Sweden: Results of a Population-Based Survey

**DOI:** 10.3390/medicina59020235

**Published:** 2023-01-26

**Authors:** Gloria Macassa, Katarina Wijk, Mamunur Rashid, Anne-Sofie Hiswåls, Chanvo Daca, Joaquim Soares

**Affiliations:** 1 Department of Public Health and Sports Science, Faculty of Occupational and Health Sciences, University of Gävle, Kungsbacksvägen 47, 80176 Gävle, Sweden; 2 EPIUnit–Instituto de Saude Publica, Universidade do Porto, Rua das Taipas 135, 4050-600 Porto, Portugal; 3 Laboratório para a Investigação Integrativa e Translacional em Saúde Populacional (ITR), Rua das Taipas 135, 4050-600 Porto, Portugal; 4 Centre for Research and Development, Uppsala University, Region Gävleborg, 80187 Gävle, Sweden; 5 Department of Occupational Health Sciences and Psychology, University of Gävle, Kungsbacksvägen 47, 80176 Gävle, Sweden; 6 Department of Public Health and Caring Sciences, Uppsala University, 75123 Uppsala, Sweden; 7 Department of Cooperation, Ministry of Health, Directorate of Planning and Cooperation, Avenida Eduardo Mondlane, Maputo P.O. Box 264, Mozambique; 8 Department of Health Sciences, Mid-Sweden University, Holmgatan 10, 85170 Sundsvall, Sweden; 9 Department of Psychology, Universidade Europeia, Estrada da Correia nº53, 1500-210 Lisbon, Portugal

**Keywords:** interpersonal violence, men, Gävleborg County, Health on Equal Terms Survey, stress, anxiety, depression

## Abstract

*Background and Objectives*: Interpersonal violence is a social and public health problem globally, and though it is related to poor health outcomes across all genders, most research has been directed towards violence against women. As a result, the health consequences of men’s victimization may be underreported and unaddressed. The purpose of this study was to assess the relationship between interpersonal violence and the psychological health outcomes of self-reported stress, anxiety, and depression among men. *Materials and Methods:* The study used data from the male sample (*n* = 2597) of the 2018 Health on Equal Terms Survey conducted in Gävleborg County in East-Central Sweden. Regression analysis was carried out to study the relationship between interpersonal violence and self-reported stress, anxiety, and depression. *Results*: The bivariate analysis showed that there was a statistically significant association between interpersonal violence and self-reported stress (OR 2.35; CI 1.45–3.81), anxiety (OR 1.54; CI 1.06–2.25), and depression (OR 2.30; CI 1.48–3.57). Controlling for other variables in the multivariate analysis removed the statistically significant relationship and reduced the odds ratios for stress (OR 1.46; CI 0.57–3.74), anxiety (OR 0.86; 0.40–1.84), and depression (OR 1.40; CI 0.67–3.32) respectively. *Conclusions*: The study found that interpersonal violence among men was associated with stress, anxiety and depression which was largely explained by demographic, socioeconomic, and health/behavior-related factors. The findings suggest the need for longitudinal studies to assess causal links between male victimization and psychological health outcomes at the county level.

## 1. Introduction

Interpersonal violence (IPV) is a social and public health problem globally [1,2,3,4] and is related to poor health outcomes across all genders [5,6,7]. It is defined as the “intentional violence between individuals either in the family or [in the] community in the form of neglect, psychological or emotional, sexual, or physical violence” [8,9]. In the family, interpersonal violence occurs at home (e.g., intimate partner violence/domestic violence and child maltreatment) [5]; in the community it occurs between strangers (e.g., sexual violence committed by an unknown person). Furthermore, IPV includes violence that occurs in institutions such as schools and workplaces [5]. Although interpersonal violence (and specifically intimate partner violence) is known to have social and public health consequences for both men and women, most research is directed towards violence against women. It is argued that this is the case because men are less likely to admit to or report violence perpetrated against them because of possible embarrassment or fear of being ridiculed, as well as lack of support services aimed specifically at men [10]. Others point to lack of data on male victimization compared with data on violence against women [11]. 

The prevalence of violence against men varies across countries and samples. For instance, in the US, Hines and Douglas report that 12% of intimate partner violence is committed against men [12]. In Germany, a nationwide survey by the Federal Criminal Police found that in 2018, 18% of men experienced violence [13]. In another study of domestic violence against men, Kolbe and Büttner reported that, among the reviewed studies, the prevalence of violence against men ranged from 3.4% to 20.3% for physical violence, and that these men were also violent against their partners [14]. Furthermore, the same report found that 10.6%–40% had been abused or maltreated during their childhood [14]. 

In Sweden, surveys have found presence of interpersonal violence against men. A study by Lövestad and Krantz found that eleven per cent of men (compared with eight percent of women) had been exposed to physical assault in the past 12 months, while women were more exposed to sexual coercion [15]. In the same study, 37% of men and 41% of women reported exposure to controlling behaviors [15]. In 2014, a report from the Swedish National Council for Crime Prevention found that, in 2012, the prevalence of domestic violence was almost similar for both genders, 6.7% for men and 7% for women [16]. Moreover, another study reported that 5% of men and 14% of women reported exposure to physical violence as well as threats of physical violence by a current or previous partner at ≥18 years of age [17]. 

Globally, including in Sweden, the consequences of IPV (for men and women alike) include increased incidence of depression, anxiety, post-traumatic stress disorder (PTSD), suicide, cardiovascular disease, premature mortality, and health behavior change (e.g., increased alcohol consumption) [18,19,20,21,22]. In Sweden, as elsewhere, much of the violence research has been carried out among women and less in men. An investigation that examines the associations between IPV and psychological health outcomes of stress, anxiety, and depression among men in East-Central region of Sweden is needed to help identify health programmatic needs. The present study addressed this timely need while also contributing to the extant literature about IPV among men. 

## 2. Materials and Methods

### 2.1. Data and Sample Description 

This study is based on secondary data from the Gävleborg Health on Equal Terms Survey (HET) 2018 [23]. The HET is a cross-sectional survey with a self-administered postal questionnaire. The 2018 survey used a randomly stratified sample (by area); the initial sample was 12,000 persons aged 16–84 living in Gävleborg County, in East-Central Sweden. A total of 5599 individuals returned the questionnaire, which gave a response rate of forty-three percent. For this study, only the male sample was analyzed (*n* = 2597). Questions were asked about the individual’s background, health, self-reported diseases or symptoms of diseases, housing, leisure, long-standing illness, social relations, political activity, finances, employment, the work environment, safety, security, and violence. The questions for the HET surveys in general have been validated and refined since its inception in 2004.

The 2018 HET survey was carried out by Statistics Sweden for the Public Health Agency of Sweden and Gävleborg Region. More details about the survey can be found elsewhere [23].

### 2.2. Variable Measurement 

#### 2.2.1. Outcome Variables

The outcome variables in the study are self-reported stress, self-reported anxiety, and self-reported depression. Self-reported psychological health outcomes were measured using the variables stress, anxiety, and depression. In the survey, participants were assessed using the questions: (a) “Do you currently feel stressed (tense, restless, nervous, uneasy, or unable to concentrate)?” Answers were “Not at all”, “To some extent”, “Quite a lot” and “Very much”; (b) “Do you have any worry or anxiety?” Possible answers were “No”, “Yes, mild symptoms” and “Yes, severe symptoms”; and (c) “Have you ever been diagnosed with depression by a doctor?” Answers were “No, never”, “Yes, more than 12 months ago”, and “Yes, in the past months”. For each of the variables, a dichotomous variable was created to distinguish those with stress, anxiety, and depression from those without.

#### 2.2.2. Independent Variables

(a) Main independent variable (main exposure)

Interpersonal violence is the main exposure in the study. In the survey, violence was measured using the following two questions, “Have you in the past 12 months been exposed to physical violence?” and “Have you in the past 12 months been exposed to threat of violence that was so severe that you got afraid?”. Individuals who answered “yes” to either one or both questions about violence were classified as victims of violence. Because of small numbers all types of violence were combined into a new variable called “any type of interpersonal violence (any IPV)”.

(b) Other independent variables (covariates)

Age was categorized into age groups: 18–29, 30–44, 45–64, and 65–84 years.

Marital status: In the survey, marital status was defined as married, unmarried, divorced, or widowed.

Self-reported health: Respondents were asked, “How do you evaluate your general health status?” with the options poor and very poor, fair, good, and very good. For this study, the answers were dichotomized. Respondents who answered fair, bad, or very bad were classified as having poor health and those who answered very good or good were classified as having good health. 

Education level: Information about the respondents’ education level was assessed from Statistics Sweden’s LISA database from 2010 and grouped using the Swedish standard classification of education SUN 2000 [24]. The original variable is classified into several groups from lowest to highest: primary school fewer than 9 years; primary school 9 or 10 years; upper secondary school more than 2 years and maximum 3 years; upper secondary school maximum 11–12 years; higher or further education less than 3 years; higher or further education 3 years or more; postgraduate study. Three groups were created for the analysis: primary school or similar; secondary school or similar; and university or similar.

Socioeconomic status: Based on the European Socioeconomic Group (ESeG) classification, a variable with three levels was constructed: high socioeconomic status (higher and lower white-collar workers); middle socioeconomic status (medium-skilled and low-skilled workers); and low socioeconomic status (blue-collar and unskilled workers). 

Individual annual income after taxation (individual annual disposable income in thousands of SEK) was divided into quartiles, with Q1 being the lowest and Q4 the highest income group: Q1 = ≤144,000 SEK; Q2 = 145,000–214,000 SEK; Q3 = 215,000–294,000 SEK; and Q4 ≥295,000 SEK.

Social support: In the survey the following question was asked: “Do you have one or more persons who can give you support when you have personal problems or a crisis in your life?” Possible answers were “yes, always”, “yes, most of the time”, “no, not most of the time”, and “no, never”. A dichotomous variable was created classifying those with social support (“yes, always” and “yes, most of the time”) from those without.

Practical support: Practical support was derived from the question, “Can you get help from someone or some people if you have practical problems or are ill (e.g., get advice, borrow things, get help with food shopping, repairs, etc)”? Possible answers were “yes, always”, “yes, most of the time”, “no, not most of the time” and “no, never”. A new variable was created dividing those with practical support from those without.

Economic strain: In the survey, respondents were asked, “In the last 12 months, have you ever had difficulty in managing the regular expenses for food, rent, bills, etc?” Possible answers were “no”, “yes, once”, and “yes, more than once”. A dichotomous variable was created dividing those with economic strain (“yes, always” and “yes, more than once”) from those without.

Risk consumption of alcohol: This was assessed by the following three questions: (a) “How often have you drunk alcohol in the past 12 months?”; (b) “How many ‘glasses’ [an example was given] do you drink on a typical day when you drink alcohol?”; and (c) “How often do you drink six ‘glasses’ or more on the same occasion?” A new dichotomic variable was created for this study and respondents were categorized as having risky alcohol consumption or as having no risky alcohol consumption.

Long-standing illnesses: In the survey, respondents were asked “Do you have any long-term illness, any problems following an accident, any reduced physical function, or any other long-term health problem?” Answers were “yes” and “no”.

#### 2.2.3. Statistical Analysis

The statistical analysis was carried out using IBM SPSS Statistics 27.0 [25] and included descriptive and logistic regression analyses. The regression analyses (bivariate and multivariate) were performed to assess the relation between any IPV and the three psychological health outcomes (stress, anxiety, and depression) across four different models. Model I pertained to bivariate analysis while Models II, III, and IV to multivariate analyses. 

Model I analyzed only the association between any IPV and stress, anxiety, and depression. In Model II, age and marital status were added to the analysis. Model III included, besides the variables already listed for Model I and II, socioeconomic variables such as education, socioeconomic status, income, economic strain, social support, and practical support. Lastly, in Model IV, health, and lifestyle variables such as self-reported health, long-standing illness, and risk consumption of alcohol were included. Missing values were excluded from the analysis. Furthermore, there was no collinearity among the variables included in the regression analysis. 

All results are presented as odds ratios (ORs) with 95% confidence intervals (CIs). 

## 3. Results

In the prior 12 months, 124 men (4.8%) in the county experienced IPV. In addition, 8.9% (*n* = 230), 28% *(n* = 726), and 11.8% (*n* = 306) reported having stress, anxiety, and depression, respectively (see Table 1). Out of the 124 men who were exposed to violence in the past 12 months, 93 experienced psychological violence and 31 reported physical violence (seven at home, 15 in entertainment venues, two on public transport, five in others’ residences, and eight in unspecified places). None reported both types of violence.

Results of the bivariate analysis across the three outcomes (stress, anxiety, and depression) showed a statistically significant association with exposure to any IPV (see Table 2, Table 3 and Table 4). For SRS, men who were exposed to any type of IPV, compared with their counterparts who were not, had an OR of 2.35 (CI 1.45–3.81) (Model I, see Table 2); for SRA, these figures were OR 1.54 (CI 1.06–2.25) (Model I, see Table 3) and for SRD, OR 2.30 (CI 1.48–3.57) (Model I, see Table 4). 

In the multivariate analysis of the association between IPV and stress (Models II, III, and IV, see Table 2), the statistically significant association disappeared after controlling for socioeconomic factors (Model III, see Table 2). There was also an overall reduction in the odds of stress from 2.35 (CI 1.45–3.81) to 1.46 (CI 0.57–3.74) (Model I and IV, see Table 2). In addition, in Model IV, men who had reported violence had statistically significant odds ratio of having poor self-rated health, with an OR of 3.81 (CI 2.29–6.36) (Model IV, see Table 2).

Regarding the statistical association between IPV and anxiety, the significance disappeared after controlling for the demographic variables age and marital status (Model II, see Table 3) in the multivariate analyses. In that same model, victimized single and divorced men had statistically significant odds of having anxiety of 1.49 (CI 1.20–1.85) and 1.20 (CI 1.10–2.10), respectively (Model II, see Table 3). Furthermore, victimized men with no social and practical support or risky alcohol behavior had higher odds of having anxiety (Model III, see Table 3). Similarly, higher odds of having anxiety were found in victimized men who self-reported poor health (Model IV, see Table 3). Overall, the odds of anxiety reduced from 1.54 (CI 1.06–2.25) to 0.86 (CI 0.40–1.84) (Model I and IV, see Table 3).

In the results of the multivariate analysis, the statistically significant association between IPV and depression disappeared after adjusting for socioeconomic factors (Model III, see Table 4). In that model, victimized men with no practical support had enhanced odds of depression of 2.41 (CI 1.13–5.14) compared with those who had practical support (Model III, see Table 4). Moreover, in Model IV, victimized men who reported poor health had odds of depression of 2.44 (CI 1.51–3.95) (Model IV, see Table 4).

## 4. Discussion

This study found that, among the surveyed male population sample in Gävleborg County, there was a prevalence of IPV of 4.8%, which constituted mainly psychological violence. Furthermore, the results of the study found that there was a statistically significant association between IPV and stress, anxiety, and depression, respectively. However, the statistical significance disappeared after controlling for demographic, socioeconomic, and health/behavior-related factors. 

The prevalence of IPV in this study is lower to that found in the 2012 HET for the entire country, in which 4% of men reported having been exposed to physical violence and 5% to threats of violence (psychological violence) [26]. Lövestad & Krantz, however, and other Swedish studies carried out by the Swedish National Council for Crime Prevention reported a higher prevalence of male IPV, of 11% and 6.7%, respectively [15,16].

Findings of this study also showed that male victims reported more psychological than physical abuse, which has been suggested to be the most common IPV type by other authors [27,28,29]. In the Netherlands, Drijber et al.’s study reported that 92 men (out of 372) suffered emotional violence and 32 suffered physical violence [27]. One study, conducted in Canada, found that almost 10.1% of men in the studied sample suffered psychological abuse [29]. Psychological violence encompasses a group of behaviors such as insults, belittling, intimidation (e.g., through destroying things), threats of harm and, in some cases of domestic violence, threats of taking their children away [30]. According to some authors, psychological/emotional violence is related to acts, threats, or coercive tactics that have an intention to humiliate, degrade, or undermine a person’s self-worth or self-esteem, to control and/or isolate them [30,31]. However, other studies have reported contrary results from their samples where physical violence was more prevalent than psychological violence. For instance, in a study carried out in Milan, Italy, it was found that many studied men were victims of physical abuse, followed by psychological violence and stalking [32]. A study from São Paolo, Brazil, found that during the 8 years analyzed, there was a high prevalence of physical violence by people known by the victims (e.g., partners), especially among young men [33].

Regarding the statistical association between IPV and the three psychological outcomes stress, anxiety, and depression, our results are in line with those reported elsewhere in studies using various samples of abused men [34,35,36]. Additionally, in the US, evidence has been found relating IPV with physical and mental health outcomes [34,37]. Others have found associations between IPV and PTSD, depression, suicidal ideation, and high blood pressure [37,38,39,40,41,42,43,44,45,46,47,48]. Furthermore, IPV in men has been related to substance use, alcohol use, smoking, and antisocial behavior, all of which are associated with negative health [44,46,49,50,51,52,53,54,55]. For instance, in a recent review of evidence of IPV against men, findings indicated that male victims of IPV also reported giving up their preferred hobbies, and missed work, became unemployed, and were likely to withdraw from family and friends [56]. 

In the regression analyses, the statistically significant relationship between IPV and stress, anxiety and depression disappeared after controlling for covariates, meaning that the covariates may mediate the observed association. In the association between IPV and anxiety, men who were single or divorced (Model II, see Table 2) and those with no social or practical support (Model III, see Table 3) had statistically significant odds of self-reporting anxiety. Similarly, in Canada, Scott-Storey and colleagues reported an increased prevalence of general anxiety disorder among men with a history of lifetime violence, compared with the general population [57]. Regarding the association between IPV and depression, men who had no practical support had increased odds of having depression (Model III, see Table 4). In samples of women (which are the most studied samples so far), having social and practical support has been associated with reduced risk of adverse psychological outcomes [58]. Additionally, when studying the relationship between any IPV and stress, anxiety, and depression, compared with good health, men reporting poor health had statistically significantly increased odds of stress (Model IV, see Table 2), anxiety (Model IV, see Table 3) and depression (Model IV, see Table 4). Other studies have reported an association between IPV and poor self-reported health [59]. 

As already mentioned above, male victimization has been neglected compared with victimization in women [60]. It is argued that there is a need to understand the role played by gender and masculinity perceptions and its effects on how IPV is experienced by men [44]. For instance, Nybergh et al. [44] suggested that it is important to understand that violence is seen differently by men and women given the fact that “gender is a pervasive structure that can be affected both by expression and [by] experiences of IPV” (p. 199). Others, too, have pointed out that gender accounts for differences regarding views and experiences of violence between men and women, as well as among men in response to IPV [35,61,62]. Furthermore, it has been argued that men experience social pressure to be in line with the hegemonic masculine norms, which, if deviated from, may put them at risk of violence [63]. According to Morgan and colleagues, in situations of IPV, pressure to fit in with and adhere to dominant gender ideals not only affects men’s sense of self but also their own and others’ appraisal and identification of the violence they experience [64]. For instance, one study reports that men who were victims of IPV took responsibility for their female partners’ abusive acts and to be a “good partner” and did not react to physical attacks to uphold what it meant to be a man [65]. Another study that investigated experiences of victimization among men suggests that society in general does not endorse the idea that men could be victims of female-perpetrated violence [66]. Some authors put forward the notion that when women perpetrate violence against men, it is not always perceived as abusive by the men themselves [67,68,69]. The argument here is that gender socialization may cause men to minimize or trivialize their experiences of IPV [62,70], but may also hinder them from disclosing their IPV experiences and from taking the decision to seek help [48,64,71,72]. Several studies carried out elsewhere also indicate that men avoid seeking help after victimization for fear of being ridiculed, because they feel ashamed, and ultimately for fear of being considered the initiator of violence rather than the victim [48,73,74,75,76,77,78]. 

As elsewhere, in Sweden most of the research has centered on women’s violence; however, there is also growing interest in male victimization. In a Swedish qualitative study of victimized men, it was found that respondents opposed full victimhood and distanced themselves from a victim role, but simultaneously wanted a status that recognized their experiences [79]. Importantly, the victimized men in the study pointed out that they experienced double victimization, first by their female partners and second by society, which viewed their victimization experiences as taboo [79]. 

The abovementioned qualitative results elucidate the potential complexities associated with male experiences of victimization even in a strong welfare state such as Sweden. Therefore, longitudinal, and qualitative studies carried out at county level can help to capture other factors that might be important in prevention and that are entrenched in local perceptions of male victimization. 

Findings of this study point to the need to a great attention to men who might seek health-care due to mental-ill health in the county, as it might be related to IPV. This is because available evidence suggests that males underutilize mental health services due to barriers both at the personal level (e.g., masculinity and low mental health literacy) as well as those associated with health care providers (e.g., lack of trust in personnel working in mental services or perceived personnel bias that favors women) [80,81,82]. Moreover, there is a need for primary services (at the county level) to screen for violence against men as well as give training to physicians and nurses to improve the identification, documentation, and referral of male patients experiencing abuse. This has been found to have an effect elsewhere [83]. A study by Williamson et al. reported an increased identification of male domestic violence after an intervention that trained general practice-based doctors and nurses [83].

### Strengths and Limitations

A major strength of this study is that the sample derives from a large dataset, collected across the entire county. In addition, the entire HET survey series (and the 2018 survey) uses validated instruments that are constantly reviewed and validated by Statistics Sweden and the Public Health Agency of Sweden [23,84]. Additionally, as previously indicated, this is the first study on male violence in the county, which is hoped to provide some insights on the prevalence as well as the impact of male victimization on mental health-related outcomes. 

However, the study has important limitations. Although it is cross-sectional in nature and the analyses only offer support for associations among the studied variables (the main exposure and the three outcomes), these associations may not be causal. Moreover, the study response rate was around forty-three percent, which is in line with the progressive decline of response rates in population-based surveys in Sweden [85] and internationally [86,87]. Some argue that non-responding groups are likely to be those with poor health [88,89,90], although we believe that this has not affected the present results. 

Nonetheless, Statistics Sweden used weightings to estimate prevalence at the population level. The weightings were performed using information from registers of the total population of Gävleborg County. Furthermore, register data were used for calibration of non-response bias for various groups of individuals in the county sample [91]. In the study, it was not possible to identify exactly in which context respondents were exposed to psychological violence as they did not voluntarily disclose that information in the survey. Lastly, this study was not able to measure social desirability bias. This bias occurs when respondents give answers that look good to others, thus concealing their true experiences and opinions [92]. However, studies carried elsewhere have found that men’s reporting of IPV as well as self-reported mental health has been affected by social desirability bias (due to an array of factors including stigma, masculinity, etc.) [92,93,94]. However, others have reported social desirability bias in which masculinity was not the main explanatory mechanism [93].

## 5. Conclusions

The present study found a statistically significant association between interpersonal violence and stress, anxiety, and depression. This association was largely explained by demographic, socioeconomic, and health/behavior-related factors. There is a need for longitudinal studies to assess causal links between male victimization and psychological health outcomes at the county level. Furthermore, qualitative research is warranted to obtain a deeper insight into the experiences and perceptions of victimized men to have a better preventive strategy that may differ from that for female victims.

## Figures and Tables

**Table 1:** Characteristics of the male sample, Gävleborg County Health in Equal Terms Survey 2018.

Variable	(n = 2597)	%
IPV (any type)		
No	2433	93.7
Yes	124	4.8
Missing	40	1.5
Self-reported health		
Good	1734	66.8
Bad	821	31.6
Missing	42	1.6
Self-reported stress		
No	2348	90.4
Yes	230	8.9
Missing	19	0.7
Self-reported anxiety		
No	1836	70.7
Yes	726	28.0
Missing	35	1.3
Self-reported depression		
No	2261	87.1
Yes	306	11.8
Missing	30	30
Age		
18–29	192	7.4
30–44	302	11.6
45–64	736	28.3
65–84	1271	48.9
Missing	96	3.7
Marital Status		
Married	1245	47.9
Single	937	36.1
Divorced	326	12.6
Widowed	87	3.4
Missing	2	0.1
Education		
Primary and similar	1385	53.3
Secondary and similar	846	32.6
University and similar	366	14.1
European socio-economic group		
High social class	330	12.7
Middle social class	324	12.5
Working class	607	23.4
Missing	1336	51.4
Individual disposable income		
Q < 144	490	18.9
Q2 144–214 Th	641	24.7
Q3 214–294	608	23.4
Q4 > 294	845	32.5
Missing	13	0.5
Social support		
Yes	2217	85.4
No	349	13.4
Missing	31	1.2
Practical support		
Yes	2418	93.1
No	155	6.0
Missing	24	0.9
Economic strain		
No	2311	89.0
Yes	136	5.2
Missing	150	5.8
Risky alcohol behavior		
No	2141	82.4
Yes	404	15.6
Missing	52	2.0
Long standing-illnesses		
No	1426	54.9
Yes	1105	42.5
Missing	66	2.5

**Table 2:** Odds ratios (ORs) with 95% confidence intervals (CIs) for the relationship between interpersonal violence (IPV) and stress among men, according to the Gävleborg Health on Equal Terms Survey 2018.

Variable	Model I	Model II	Model III	Model IV
	OR (95% CI)	OR (95% CI)	OR (95% CI)	OR (95% CI)
IPV (any type)				
No	1	1	1	1
Yes	2.35 (1.45–3.81) ***	1.81 (1.08–3.03) ***	1.52 (0.66–3.50)	1.46 (0.57–3.74)
Age, yrs				
18–29		1	1	1
30–44		0.82 (0.48–1.38)	0.55 (0.17–1.80)	0.50 (1.44–1.76)
45–64		0.41 (0.52–1.37)	0.45 (014–1.49)	0.36 (0.11–1.22)
65–84		0.31 (0.18–1.53)	0.24 (0.67–1.83)	0.19 (0.53–1.69)
Marital status				
Married		1	1	1
Single		1.24 (0.88–1.75)	1.04 (0.65–1.72)	0.91 (0.64–1.30)
Divorced		1.70 (1.09–2.64)	0.85 (0.39–1.88)	0.80 (0.31–1.16)
Widowed		0.60 (0.14–2.52)	1.20 (0.14–9.91)	0.96 (0.21–1.01)
Education				
Primary and similar			1.28 (0.65–2.54)	1.17 (0.57–2.39)
Secondary and similar			0.98 (0.54–1.80)	1.01 (0.54–1.90)
University and similar			1	1
European Socioeconomic Group classification				
High social class			1	1
Middle social class			0.56 (0.30–1.04)	0.61 (0.32–1.16)
Working class			0.57 (0.31–1.04)	0.56 (0.30–1.03)
Individual disposable income, thousands SEK				
Q1 ≤ 144			0.56 (0.17–1.88)	0.37 (0.95–1.45)
Q2 = 144–214			0.85 (0.39–1.84)	0.77 (0.34–1.73)
Q3 = 215–294			0.61 (0.35–1.08)	0.61 (0.34–1.10)
Q4 ≥ 295			1	1
Social support				
Yes			1	1
No			1.52 (0.86–2.69)	1.29 (0.71–2.35)
Practical support				
Yes			1	1
No			4.66 (2.11–9.85)	4.19 (1.91–9.17)
Economic strain				
No			1	1
Yes			2.02 (0.98–4.17)	1.43 (0.65–3.14)
Risky alcohol behaviour				
No				1
Yes				1.07 (0.62–1.83)
Long-standing illnesses				
No				1
Yes				0.86 (0.53–1.42)
Self-reported health				
Good				1
Poor				3.81 (2.29–6.36) ***

*** *p* < 0.001; Model I: IPV and SRS only; Model II: Model I + age and marital status; Model III: Model I + Model II + socioeconomic variables; and Model IV: Model I + Model II + Model III + health/behavior-related variables.

**Table 3:** Odds ratios (ORs) with 95% confidence intervals (CIs) for the relationship between interpersonal violence (IPV) and anxiety among men, according to the Gävlebörg Health on Equal Terms Survey 2018.

Variable	Model I	Model II	Model III	Model IV
	OR (95% CI)	OR (95% CI)	OR (95% CI)	OR (95% CI)
IPV (any type)				
No	1	1	1	1
Yes	1.54 (1.06–2.25) ***	1.38 (0.93–2.05)	1.06 (0.57–1.97)	0.86 (0.40–1.84)
Age, yrs				
18–29		1	1	1
30–44		1.15 (0.78–1.71)	1.47 (0.56–3.90)	2.34 (0.73–7.48)
45–64		0.96 (0.67–1.38)	1.51 (0.57–4.01)	2.08 (0.65–6.57)
65–84		0.88 (0.61–1.27)	1.12 (0.40–3.05)	1.59 (0.48–5.23)
Marital status				
Married		1	1	1
Single		1.49 (1.20–1.85) ***	1.14 (0.83–1.58)	1.01 (0.70–1.46)
Divorced		1.20 (1.10–2.10) ***	1.21 (0.75–1.96)	1.36 (0.80–2.32)
Widowed		1.10 (0.62–1.93)	1.13 (0.30–4.27)	1.30 (0.31–5.38)
Education				
Primary and similar			1.01 (0.64–1.61)	0.96 (0.56–1.59)
Secondary and similar			1.03 (0.60–1.57)	1.17 (0.73–1.87)
University and similar			1	1
European Socioeconomic Group classification				
High social class			1	1
Middle social class			0.88 (0.58–1.34)	0.97 (0.61–1.55)
Working class			1.01 (0.67–1.52)	1.16 (0.73–1.85)
Individual disposable income, thousands SEK				
Q1 ≤ 144			1.28 (0.62–2.64)	1.51 (0.67–3.41)
Q2 = 145–214			1.12 (0.68–1.85)	1.05 (0.59–1.87)
Q3 = 215–294			1.02 (0.72–1.44)	1.12 (0.76–1.65)
Q4 ≥ 295			1	1
Social support				
Yes			1	1
No			1.49 (1.00–2.22) ***	1.38 (0.88–2.17)
Practical support				
Yes			1	1
No			2.07 (1.10–3.88) ****	1.02 (0.48–2.18)
Economic strain				
No			1	1
Yes			2.15 (1.25–3.71) ***	1.65 (0.85–3.09)
Risky alcohol behaviour				
No				1
Yes				1.24 (0.84–1.83)
Long-standing illnesses				
No				1
Yes				0.99 (0.70–1.40)
Self-reported health				
Good				1
Poor				2.85 (1.95–4.15) ***

*** *p* < 0.001; Model I: IPV and SRA only; Model II: Model I + age and marital status; Model III: Model I + Model II + socioeconomic variables; and Model IV: Model I + Model II + Model III + health/behavior-related variables.

**Table 4:** Odds ratios (ORs) with 95% confidence intervals (CIs) for the relationship between interpersonal violence (IPV) and depression among men, according to the Gävleborg Health on Equal Terms Survey 2018.

Variable	Model I	Model II	Model III	Model IV
	OR (95% CI)	OR (95%CI)	OR (95% CI)	OR (95% CI)
IPV (any type)				
No	1	1	1	1
Yes	2.30 (1.48–3.57) ***	2.00 (1.26–3.18) ***	1.42 (0.67–3.05)	1.40 (0.67–3.32)
Age, yrs				
18–29		1	1	1
30–44		1.59 (0.93–2.72)	1.26 (0.33–4.88)	1.41 (0.34–5.78)
45–64		1.19 (0.71–1.99)	1.01 (0.26–3.93)	0.93 (0.22–3.85)
65–84		0.86 (0.51–1.49)	0.65 (0.16–2.63)	0.57 (0.13–2.49)
Marital status				
Married		1	1	1
Single		1.24 (0.91–1.69)	0.78 (0.49–1.24)	071 (0.44–1.16)
Divorced		1.84 (1.27–2.65) ***	1.55 (0.85–2.84)	1.70 (0.90–3.23)
Widowed		1.57 (0.75–3.28)	0.87 (0.11–7.09)	1.08 (0.13–8.88)
Education				
Primary and similar			0.99 (0.52–1.90)	1.02 (0.51–2.04)
Secondary and similar			1.15 (0.66–2.03)	1.35 (0.75–2.45)
University and similar			1	1
European Socioeconomic Group classification				
High social class			1	1
Middle social class			0.83 (0.47–1.45)	0.88 (0.49–1.57)
Working class			0.64 (0.37–1.12)	0.62 (0.35–1.10)
Individual disposable income, thousands SEK				
Q1 ≤ 144			1.16 (0.41–3.28)	0.97 (0.31–3.01)
Q2 = 145–214			1.70 (0.86–3.37)	1.64 (0.80–3.35)
Q3 = 215–294			1.60 (0.98–2.61)	1.63 (0.98–2.72)
Q4 ≥ 295			1	1
Social support				
Yes			1	1
No			1.39 (0.81–2.41)	1.25 (0.71–2.19)
Practical support				
Yes			1	1
No			2.41 (1.13–5.14) ***	1.51 (0.66–3.46)
Economic strain				
No			1	1
Yes			1.12 (0.56–2.60)	1.00 (0.45–2.22)
Risky alcohol behaviour				
No				1
Yes				0.78 (0.45–1.34)
Long-standing illnesses				
No				1
Yes				1.31 (0.84–2.04)
Self-reported health				
Good				1
Poor				2.44 (1.51–3.95) ***

*** *p* < 0.001; Model I: IPV and SRD only; Model II: Model I + age and marital status; Model III: Model I + Model II + socioeconomic variables; and Model IV: Model I + Model II + Model III + health/behavior-related variables.

## Data Availability

The data presented in this study are available on request from the corresponding author. The data are not publicly available owing to restrictions in the ethical approval for this study. Questions related to the data in this study should be directed to the corresponding author.
